# Screening and selection strategy for the establishment of biosimilar to trastuzumab-expressing CHO-K1 cell lines

**DOI:** 10.1186/s13568-020-01157-6

**Published:** 2021-01-03

**Authors:** Thailin Lao-Gonzalez, Alexi Bueno-Soler, Arnelys Duran-Hernandez, Katya Sosa-Aguiar, Luis Eduardo Hinojosa-Puerta, Tays Hernandez-Garcia, Kathya Rashida de la Luz-Hernandez, Julio Palacios-Oliva, Tammy Boggiano-Ayo

**Affiliations:** 1 Process Development Direction, Center of Molecular Immunology, Playa, Havana, 11600 Cuba; 2 Animal Biotechnology Division, Center for Genetic Engineering and Biotechnology, Playa, Havana, 10600 Cuba; 3 Faculty of Biology, University of Havana, Havana, Cuba; 4 Immunotherapy Direction, Center of Molecular Immunology, Playa, 11600 Havana, Cuba; 5 CIMAB S. A, Playa, 11600 Havana, Cuba

**Keywords:** Biosimilar, Trastuzumab, CHO-K1 cells, Lentivirus, Intracellular staining, Flow cytometry

## Abstract

The high prices of biopharmaceuticals or biologics used in the treatment of many diseases limit the access of patients to these novel therapies. One example is the monoclonal antibody trastuzumab, successfully used for breast cancer treatment. An economic alternative is the generation of biosimilars to these expensive biopharmaceuticals. Since antibody therapies may require large doses over a long period of time, robust platforms and strategies for cell line development are essential for the generation of recombinant cell lines with higher levels of expression. Here, we obtained trastuzumab-expressing CHO-K1 cells through a screening and selection strategy that combined the use of host cells pre-adapted to protein-free media and suspension culture and lentiviral vectors. The results demonstrated that the early screening strategy obtained recombinant CHO-K1 cell populations with higher enrichment of IgG-expressing cells. Moreover, the measurement of intracellular heavy chain polypeptide by flow cytometry was a useful metric to characterize the homogeneity of cell population, and our results suggest this could be used to predict the expression levels of monoclonal antibodies in early stages of cell line development. Additionally, we propose an approach using 25 cm^2^ T-flasks in suspension and shaking culture conditions as a screening tool to identify high producing cell lines. Finally, trastuzumab-expressing CHO-K1 clones were generated and characterized by batch culture, and preliminary results related to HER2-recognition capacity were successful. Further optimization of elements such as gene optimization, vector selection, type of amplification/selection system, cell culture media composition, in combination with this strategy will allow obtaining high producing clones.

## Introduction

Trastuzumab is a humanized antibody (IgG1 isotype) specific for the human epidermal growth factor receptor 2 (HER2) that was generated in a Chinese hamster ovary (CHO) cell line by Genentech (Dillman 1999; Heffner et al. 2015; McDonnell 2015). This antibody is currently commercialized by Roche for the treatment of HER2-positive breast cancer in the adjuvant and metastatic setting, and HER2-positive metastatic gastric or gastroesophageal junction adenocarcinoma (Genentech 1998). The extraordinary achievements of trastuzumab in clinical setting have made history in the systematic treatment of breast cancer. Unfortunately, it is not uniformly available for routine use owing to its prohibitively high cost (Pivot and Petit 2018). The expiration of the European Union (EU) patent of trastuzumab in 2014, and the last version of US patent in 2019 (Nelson 2014) encouraged the development of biosimilars to this antibody (Curigliano et al. 2016). Biosimilars, are predicted to be 20%–30% cheaper than the reference product. For this reason it is expected to have a positive effect in terms of cost-effectiveness and increased availability and accessibility of targeted therapies (Paplomata and Nahta 2018). In order to guarantee the access of Cuban patients to this prohibitively expensive therapy, the Center of Molecular Immunology (CIM, Havana, Cuba) decided to develop a biosimilar to trastuzumab produced in CHO-K1 cells.

The use of CHO cells for therapeutic proteins production has many advantages such as the easier adaptation to grow in serum-free and/or chemically defined media, the ability to reach high cell densities in suspension culture, high product titer and similar glycosylation patterns to human cell lines (Bandaranayake and Almo 2014; Kim et al. 2012; Kuystermans and Al-Rubeai 2015a; Spearman and Butler 2015). Nowadays, platforms based on CHO cells use cells pre-adapted to grow in chemically defined, serum-free media and suspension culture to decrease time and efforts related to adaptation of the recombinant CHO cell lines (Jostock 2011; Kim et al. 2012). Additionally, these cells can be easily transduced using lentiviral vectors (LVs) (Gaillet et al. 2010; Oberbek et al. 2011). This transfection method takes advantage of LV mechanisms for stable integration within the chromosome of the cells, specifically into transcriptionally active regions. This feature makes them excellent tools for obtaining cell lines with high expression levels of target recombinant proteins (Gödecke et al. 2018; Mursi and Masuda 2018). LVs as a gene transfer method provides additional opportunities for optimization as it allows the adjustment of the virus to cell ratio (multiplicity of infection, MOI) to modulate transgene copy number and/or the percentage of infected cells (Gödecke et al. 2018; Oberbek et al. 2011).

Once the cells are transfected, the next crucial step is to identify desirable variants or clones from the heterogeneous transfectant pool (Priola et al. 2016). Several methods have been used, from automated cloning techniques such as ClonePix FL™ and Cello™ system, to manual techniques such as limiting dilution cloning (Kuystermans and Al-Rubeai 2015b).

Regarding antibodies production, several reports have demonstrated a positive correlation between productivity (volumetric or specific) and levels of mRNA or intracellular light chain (LC) or heavy chain (HC) polypeptides using flow cytometry. Despite the contrasting results on this issue (Borth et al. 1999; Chusainow et al. 2009; Dorai et al. 2006; Edros et al. 2013; Jiang et al. 2006; Lattenmayer et al. 2007; Lee et al. 2009; Park and Ryu 1994; Roy et al. 2017), it will be important to verify whether these variables could predict the expression levels of target antibodies on early stage of the cell line development process in order to reduce the time to obtain the recombinant cell lines.

In the present work we generated several trastuzumab-expressing cell lines following a different strategy than previously employed to obtain other biosimilar candidates from NS0 cells and CHO-K1 cells at CIM (unpublished data). In those cases, all the cell line development process and productivity assessments were performed in serum supplemented medium and adherent culture followed by a serum‐free suspension adaptation step. Herein, we combined the use of CHO-K1 cells pre-adapted to grow in protein-free media and suspension culture, the transduction with lentiviral vectors, an early screening step and the early adaptation of recombinant cell lines generated to the afore mentioned conditions. Furthermore, the assessment of the expression levels and screening of producing cell lines was done in a setting more similar to that of a final production process. Our results suggest that the measurement of intracellular HC polypeptide by flow cytometry could be used to predict the expression levels of monoclonal antibodies on early stages. This would be an advantageous approach to select precursor cell pools enriched with high producing clones. In addition, biosimilar to trastuzumab-expressing clones were characterized by a batch culture. Finally, the capacity of the recombinant antibody to recognize the HER2 molecule expressed in tumor cell lines was assessed by flow cytometric techniques.

## Materials and methods

### Cell lines and cell culture conditions

Human embryonic kidney 293T cells (HEK-293T) were used as packing cell line for the production of LVs. These cells were grown in DMEM/F12 medium (Gibco, USA) with 5% FBS (HyClone, GE Healthcare, USA) (DMEM/F12-FBS) at 37 °C in 5% CO_2_.

CHO-K1 cells previously adapted to grow in suspension culture and chemically-defined, protein-free media were grown in CP-CHO medium (Merck, Germany) supplemented with 3 g/L of HyClone Cell Boost 5 (CB5) (GE Healthcare, USA) (CP-CHO-CB5). This is a chemically-defined, protein-free medium. Cultures were shaken at 120 rpm and cultivated at 37 °C in 5% CO_2_.

The medium DMEM/F12-FBS was used for cell transduction and cloning procedures. Recombinant cell lines obtained were directly re-adapted to CP-CHO-CB5 medium and suspension culture or by step-wise decrease of FBS from 1% to 0.5%, and 0%. The drug Geneticin (G418) (Gibco, USA) was used as selection agent at a final concentration of 0.6 mg/mL.

For HER2-recognition assays were used the following tumor cell lines. SKBR3 cells (HER2 + , CD20 −) were cultured in McCoy’s 5A medium (Gibco, USA) with 10% FBS. SKOV3 cells (HER2 + , CD20 −) were cultured in DMEM/F12-FBS medium with 10% FBS. Ramos cells (HER2−, CD20 +) were cultured in RPMI medium (Gibco, USA) with 10% FBS. All the cells were incubated at 37 °C in 5% CO_2_.

All cell lines used in this work were obtained from ATCC.

### Plasmids

**Fig. 1:**
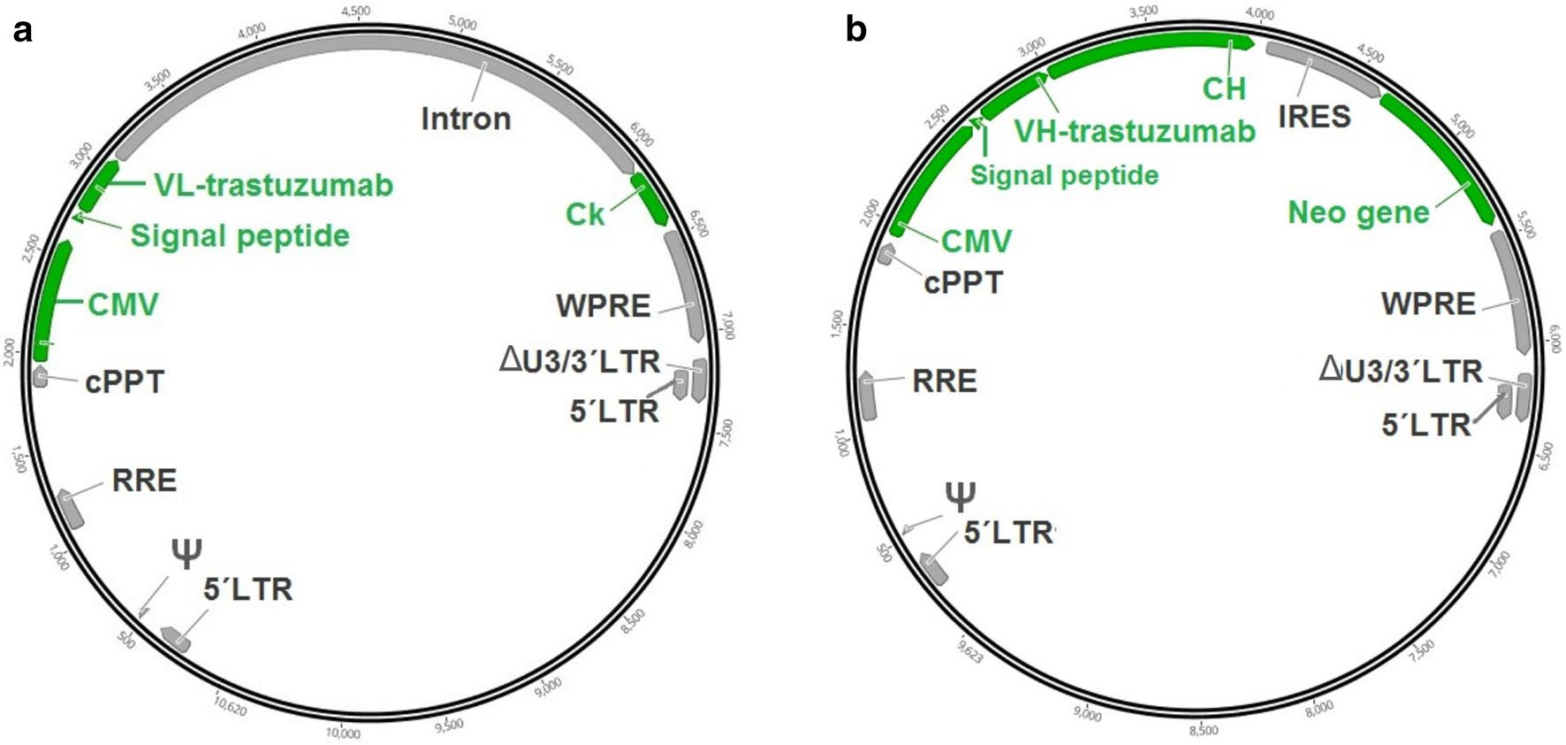
Schematic diagram of the LV transfer plasmids used. **a** pLW-CMV-trastuzumab LC and **b** pLV-CMV-trastuzumab HC-IRES-Neo, encoding light chain (LC) and heavy chain (HC) of trastuzumab, respectively. 5´LTR: HIV-1 truncated 5′ long terminal repeat. Ψ: HIV-1 psi packaging signal. *RRE* Rev response element, *cPPT* central polypurine tract, *CMV* human cytomegalovirus promoter, *VL* variable region of LC, *Ck* constant region of LC (kappa), *VH* variable region of HC, *CH* constant region of HC, *IRES* internal ribosome entry site, *Neo gene* neomycin phosphotransferase gene, *WPRE* woodchuck hepatitis virus posttranscriptional regulatory element. ΔU3/3´LTR: HIV-1 truncated 3′ long terminal repeat

The plasmids used in this work correspond to the third generation HIV-1-based LV packaging system and included three helper plasmids (Invitrogen, USA): (1) pLP1 (contains gag and pol genes), (2) pLP2 (contains rev gene) and 3) pLP VSV-G (contains VSV G glycoprotein gene). For trastuzumab expression, LC and HC, carried by different lentiviral plasmids, were expressed by the human cytomegalovirus promoter (CMV). Trastuzumab gene sequences were previously obtained from publicly available databases (DrugBank and patent 5,821,337). The plasmid pLW-CMV-trastuzumab LC (courtesy of Chimeric Protein Group of CIM) bears the gene encoding for trastuzumab LC and no selection marker (Fig. 1a). To construct plasmid pLV-CMV-trastuzumab HC-IRES-Neo (Fig. 1b), variable region of trastuzumab HC gene was amplified by PCR from pLW-CMV-HCH-IRES-AcGFP1 (courtesy of Chimeric Protein Group of CIM), which bears variable region of trastuzumab HC, obtaining fragment 1. The forward and reverse oligonucleotide primers were 5´-TACTTAGGATCCCACCATGGAATGCAGC-3´ (THA1) and 5´-TGGGCCCTTGGTGCTAGC-3´ (THA2). In parallel, the constant region of HC gene was amplified by PCR from pFUSEss-CHIg-hG1 (InvivoGen, USA). The forward and reverse oligonucleotide primers were 5´-GCTAGCACCAAGGGCCCA-3´ (THA3) and 5´- GTACAGCTCGAGTCATTTACCCGGAGACAGGGA-3´ (THA4). Finally, fragments 1 and 2 were joined by an overlapping PCR using oligonucleotide primers THA1 and THA4, to obtain the gene encoding for trastuzumab HC. The PCR product was digested with *Bam*HI and *Xho*I and ligated upstream of an IRES (internal ribosome entry site) sequence in plasmid pLV-CMV-IRES-Neo (courtesy of Chimeric Protein Group of CIM) digested with the same two restriction enzymes. This plasmid bears a gene encoding for neomycin phosphotransferase selection marker (Neo) downstream of an IRES sequence.

### Monoclonal antibodies

Trastuzumab (trade name Herceptin), a humanized mAb specific for the human HER2 molecule, was purchased from Roche (Argentine). A biosimilar candidate to trastuzumab, named 5G4 and obtained from murine NS0 myeloma cells, was provided by Development Department of CIM (Havana, Cuba).

### Quantification of human IgG-expression levels by ELISA

The human IgG-expression levels in cell culture supernatant were determined by sandwich ELISA. 96 well plates (High Binding, Costar, USA) were coated with 3 μg/mL of a goat anti-human IgG (γ chain specific) antibody (Sigma-Aldrich, USA) using coating buffer (Na_2_CO_3_/NaHCO_3_ 0.1 M, pH 9.6). After a step of incubation at 4 °C during 16 h, the plates were washed three times with washing buffer (phosphate buffered saline (PBS); Tween 20 at 0.05%, pH 7.5). The samples, diluted in blocking buffer (washing buffer and bovine serum albumin (BSA) at 0.25%), were applied to the plates and incubated at 37 °C during 1 h. Then, the plates were washed three times with washing buffer and an alkaline phosphatase (AP)-conjugated goat anti-human IgG (γ chain specific) antibody (Sigma-Aldrich, USA) was added. After another step of incubation at 37 °C during 1 h, the plates were washed again and substrate was added (5 mg of p-nitrophenyl phosphate diluted in 5 mL of diethanolamine, pH 9.8). 30 min later, the reaction was stopped with NaOH 3 M and absorption was measured at 405 nm on a microplate reader (Dialab, Austria). To quantify the expression levels, commercial trastuzumab was used as a standard (standard curve ranges from 3.9 to 125 ng/mL). Samples were analyzed in triplicate.

In addition, another type of sandwich ELISA was used, allowing detection and quantification of antibody whole molecule. In this case, the samples were diluted in a different blocking buffer (washing buffer and 5% FBS) and it was used a horse-radish peroxidase (HRP)-conjugated goat anti-human kappa light chain antibody (Sigma-Aldrich, USA). The substrate was 5 mg of o-phenylenediamine dihydrochloride (OPD) in 10 mL of citrate–phosphate buffer (pH 4.2) and 20 μL of H_2_O_2_ at 30%. Absorption was measured at 490 nm on a microplate reader (Dialab, Austria). Samples were analyzed in triplicate.

### Production and quantification of LVs

LVs were produced by transfection of HEK-293T using lineal PEI (Sigma-Aldrich, USA) as previously described (Toledo et al. 2009) with some modifications. HEK-293T cells were cultured in a 75 cm^2^ T-flask in DMEM/F12-FBS medium until cells reached up to 70–80% confluence. The cells were co-transfected with one of the lentiviral transfer plasmids (pLW-CMV-trastuzumab LC or pLV-CMV-trastuzumab HC-IRES-Neo) and helper plasmids: pLP1, pLP2 and pLP VSV-G at a ratio of (2:1:1:1) (w:w:w:w) for 30 μg of total DNA. Prior to transfection, cell culture supernatant was removed, the cells were washed with DMEM/F12 medium and 10 mL of this medium was added. In parallel, a mix of DNA, PEI and DMEM/F12 medium was prepared and added directly to the cells. After 6 h of incubation at 37 °C in the presence of 5% CO_2_, 1 mL of FBS was added to the culture and the supernatant was harvested at 72 h post-transfection. The cell culture supernatant was centrifuged at 290 *g* for 5 min, filtered (0.45 mm membrane) and stored to 4 °C for immediate use, or − 80 °C for long periods of time. The Lenti-X™ Concentrator kit (Clontech, USA) was used to purify the LVs following manufacturer’s instructions. The LVs pellet was diluted in 200–600 μL of DMEM/F12 medium and stored at − 80 °C. An ELISA for detection of HIV p24 capsid protein (DAVIH-Ag p24, LISIDA, Cuba) was used for titration of the concentrated LVs stocks.

### Transduction of CHO-K1 cells using LVs and generation of trastuzumab-expressing cell pools, mini-pools and oligoclones

Day prior transduction, 5 × 10^3^ CHO-K1 cells were seeded in 96 well plate using DMEM/F12-FBS medium and incubated at 37 °C in 5% CO_2_. After 16 h, transduction was performed by incubating LVs with cells in DMEM/F12 medium supplemented with 10 μg/mL of polybrene (Sigma-Aldrich, USA). For co-transduction, two different MOI ratios were used: (400:400) and (600:200) (MOI for LVs bearing LC: MOI for LVs bearing HC) (MOI-LC: MOI-HC). Eight hours post-transduction, the medium was replaced with fresh DMEM/F12-FBS medium and 0.6 mg/mL of G418 (selection medium). A second round of co-transduction was performed in the same conditions as outlined above. The cell culture supernatant was harvested at 72 h post-transduction and human IgG-expression was assessed by ELISA (antibody whole molecule).

**Fig. 2:**
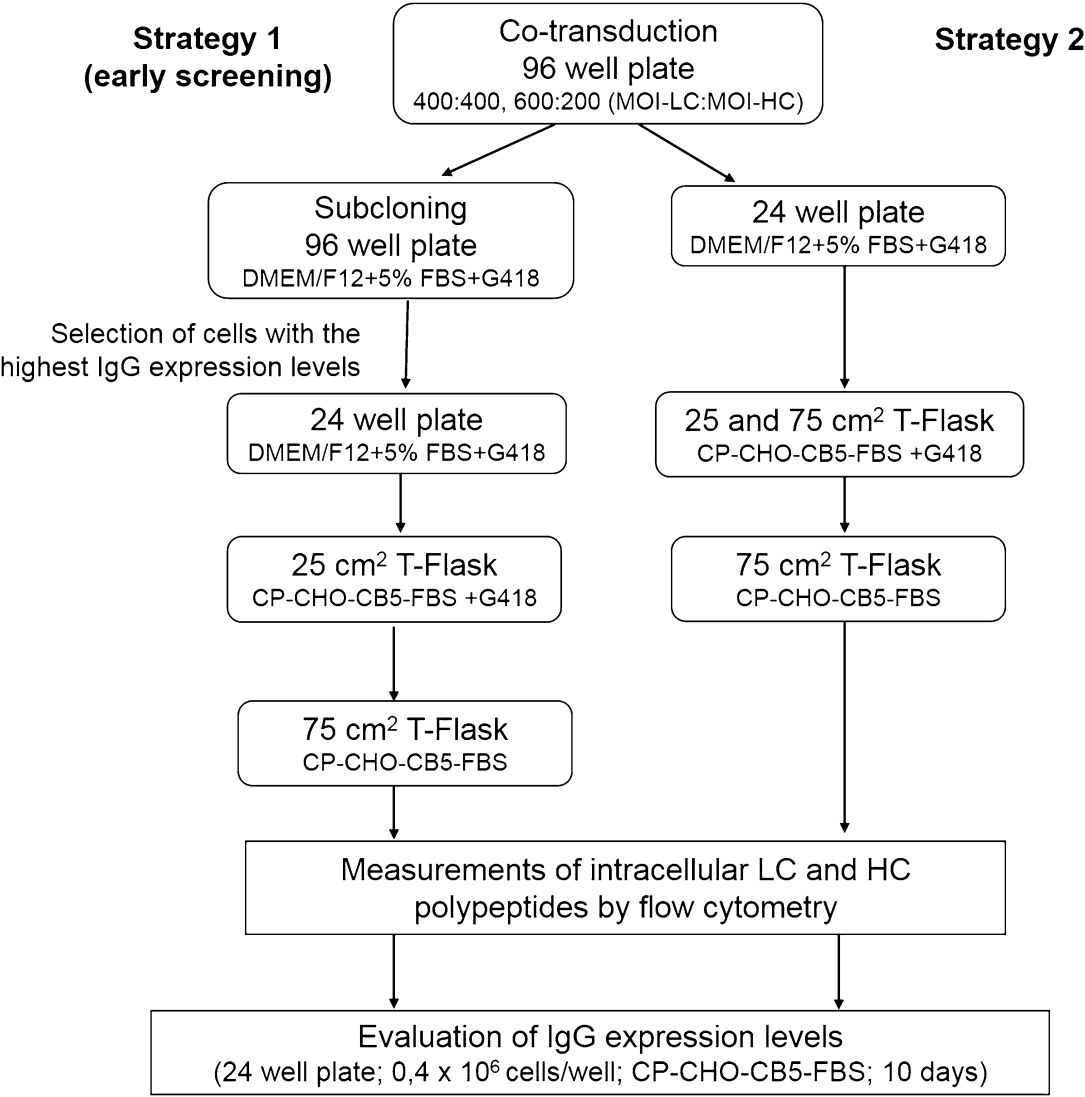
Strategies for obtaining trastuzumab-expressing CHO-K1 cells. MOI: multiplicity of infection. FBS: fetal bovine serum. CP-CHO-CB5-FBS: chemically defined, protein-free medium CP-CHO supplemented with 3 g/L of CB5 and 1% FBS. *LC* light chain, *HC* heavy chain

To obtain cell mini-pools, after a second round of transduction, cells were cloned in 96 well plate (strategy 1 or early screening) (Fig. 2). The cells were seeded in 10 plates at 100 cells/well in DMEM/F12-FBS medium and 0.6 mg/mL of G418. The plates were incubated at 37 °C in 5% CO_2_ during 10 days. The 10 cell mini-pools with the highest IgG expression levels, for each co-transduction ratio, were expanded to suspension culture in 25 cm^2^ T-flasks using CP-CHO-CB5 medium supplemented with 1% FBS (CP-CHO-CB5-FBS) and 0.6 mg/mL of G418. T-flasks were incubated in vertical position at 37 °C in 5% CO_2_ and shaking culture (120 rpm) (Infors HT, Switzerland). After 21 days under drug pressure in presence of low content of FBS (1%), cells were cultured in previously mentioned conditions without G418.

To generate cell pools, transduced cells were expanded to 24 well plate in DMEM/F12-FBS medium and 0.6 mg/mL of G418 (strategy 2) (Fig. 2). The plate was incubated at 37 °C in 5% CO_2_ until cells reached up to 90% confluence. Then, cells were expanded to suspension culture in 25 cm^2^ T-flasks in CP-CHO-CB5-FBS and 0.6 mg/mL of G418. T-flasks were incubated in vertical position at 37 °C in 5% CO_2_ and shaking culture (120 rpm) (Infors HT, Switzerland). After 26 days under drug pressure in presence of low content of FBS (1%), cells were cultured as outlined above without G418.

To obtain cell oligoclones, the two cell mini-pools with the highest IgG expression levels were cloned in 96 well plates. The cells were seeded in 10 plates at 10 cells/well in DMEM/F12-FBS medium and were incubated at 37 °C in 5% CO_2_. After 10 days, cell culture supernatant was removed to quantify human IgG-expression levels by ELISA. The oligoclones with the highest IgG expression levels in this stage, were expanded to 24 well plate in CP-CHO-CB5-FBS medium in static culture at 37 °C in 5% CO_2_. Later, they were cultured in 25 cm^2^ and 75 cm^2^ T-flasks in shaking culture (120 rpm) (Infors HT, Switzerland).

### Limiting dilution cloning

The three oligoclones with the highest IgG expression levels were cloned by limiting dilution in 96 well plate. The cells were plated in 10 plates at 0.5 cells/well in DMEM/F12-FBS medium and were incubated at 37 °C in 5% CO_2_. At day 10 and 14, plates were analyzed and those wells with only one colony of cells were marked. The cell culture supernatant of these selected wells was harvested in different times, taking into account that culture medium was metabolized, and IgG expression levels were quantified by ELISA. Then, concentration of IgG to time of supernatant harvest (ng/(mL*day)) ratio was used to select clones with highest IgG expression levels. Fifty-two clones with values above 180 ng/(mL*day) were expanded, first to 24 well plate and later to 25 cm^2^ T-flasks in CP-CHO-CB5 medium. Cells were cultured directly from a medium supplemented with 5% FBS to a chemically defined, protein-free medium, without an adaptation stage by step-wise decrease of FBS.

### Assessment of IgG expression levels in cell culture supernatant in 24 well plate and shaking T-flask

To assess the IgG expression levels in cell culture supernatant in static culture, cells were seeded in 24 well plates at 0.4 × 10^6^ cells/well in 1 mL of CP-CHO-CB5-FBS medium. The experiment was performed in triplicates. Plates were incubated at 37 °C in 5% CO_2_ and after 9–10 days cell culture supernatant was removed to quantify human IgG-expression levels by ELISA.

To evaluate the IgG expression levels in cell culture supernatant in shaking culture, cells were seeded in 25 cm^2^ T-flasks at 0.4 × 10^6^ cells/mL in 10 mL of CP-CHO-CB5 medium. The experiment was performed in duplicates. T-flasks were incubated at 37 °C in 5% CO_2_ and shaking culture (120 rpm) (Infors HT, Switzerland) in vertical position. After 10 days, supernatant was removed to quantify human IgG-expression levels by ELISA.

### Assessment of intracellular LC and HC polypeptides by flow cytometry

The intracellular LC and HC polypeptides of CHO-K1 cells, wild type or antibody expressing, was measured by flow cytometry. The cells were fixed and permeabilized using ethanol, as previously described (Lee et al. 1993). Intracellular HC and LC polypeptides were stained with a fluorescein-isothiocyanate (FITC)-labeled goat anti-human IgG (γ chain specific) antibody (Sigma-Aldrich, USA) and a FITC-labeled goat anti-human kappa light chain antibody (Sigma-Aldrich, USA), respectively. Stained cells were analyzed on a Gallios flow cytometer (Beckman Coulter, USA) and data were processed with FlowJo 7.6.1 software (Tree Star Inc., USA).

### Batch culture

In order to perform batch culture, shaking Erlenmeyer flasks with 60 mL of CP-CHO-CB5 medium were inoculated with 0.3 × 10^6^ cells/mL and incubated at 37 °C in 5% CO_2_ and shaking culture (120 rpm) (Infors HT, Switzerland). The experiment was performed in duplicates. Every 24 h, samples of cell culture were collected and cell density and viability were determined by the trypan blue exclusion method. Cell culture supernatant was harvested to quantify human IgG-expression levels by ELISA (antibody whole molecule). The maximum growth rate (μmax) in h^−1^ was calculated as the slope of the following Eq. 1:

$$VCD\left( t \right) = VCD\left( {t0} \right) + \mu max \times t$$

where VCD(t0) and VCD(t) are viable cell density at times 0 and t in the exponential phase of cell growth, respectively. The specific productivity (q_IgG_) in pg/cell/day (pcd) was determined in the exponential phase of cell growth according to Eq. 2:

q_IgG_ = $\frac{1}{VCD} \times \frac{{d\left[ {IgG} \right]}}{dt}$ where VCD(t) is the viable cell density at time t (h), concentration of IgG [IgG] in μg/mL. Trapeze method was used to calculate the time integral of viable cell concentration (IVCC) (ʃXv dt) (10^6^ cells*hour/mL).

### SDS-PAGE and western blotting analysis

SDS-PAGE was performed as described (Laemmli, 1970). Cell culture supernatant samples; 2.5 μg of commercial trastuzumab (anti-HER2; Roche, Argentine) and 2.5 μg of a biosimilar candidate to trastuzumab (named 5G4) (CIM, Cuba), were prepared under reducing conditions with sample buffer (containing beta-mercaptoethanol) for one minute at 95 °C or under non-reducing conditions (sample buffer without reducing agent). In all cases, 15 μL of the cell culture supernatant sample were used and concentration of IgG was not taken into account. A Color Prestained Protein Standard (NEB, UK) was used as molecular weight marker. Samples and controls under non-reducing conditions were loaded in 7.5% SDS-PAGE gel and for reducing conditions were loaded in 12% SDS-PAGE gel. Proteins were transferred to nitrocellulose membranes (Whatman, USA) by electric field in semi-humid conditions using a Semiphor Transphor Unit (Pharmacia Biotech, USA). Antibody detection was carried out with a HRP-conjugated goat anti-human kappa light chain antibody (Sigma-Aldrich, USA) or an AP-conjugated goat anti-human IgG (γ chain specific) antibody (Sigma-Aldrich, USA). A TMB reagent for western blotting (Sigma-Aldrich, USA) and Color development kit (BIO-RAD, USA) were used, respectively, as substrate.

### HER2-recognition assay

The recognition of human HER2 by biosimilar candidate to trastuzumab contained in cell culture supernatant was evaluated by flow cytometry. Tumoral cell lines overexpressing HER2 molecule: SKBR3 and SKOV3 were used. Ramos cells (CD20 +) were also used as a negative control of HER2 expression and recognition. Cells were stained with 10 μg/mL of produced antibody contained in cell culture supernatants or commercial trastuzumab (anti-HER2; Roche, Argentine) for 30 min at 4 °C. A biosimilar candidate to rituximab (anti-CD20) antibody contained in cell culture supernatant (10 μg/mL) was added as isotype-matched control. Cells were washed with PBS and the binding of the antibodies was detected by incubation with a FITC-labeled rabbit anti-human IgG antibody (F0315, Dako, USA) for 30 min at 4 °C. Cells were analyzed on a Sysmex flow cytometer (Germany) and data were processed with FlowJo 7.6.1 software (Tree Star Inc., USA).

### Statistical analysis

The results are expressed as mean ± standard deviation (SD). When necessary, data were analyzed by Student’s T test, one-way ANOVA, Tukey test or Pearson correlation using Minitab 16.1.0 software (Minitab Inc., USA). The difference between the means was considered statistically significant at p < 0.05.

## Results

### Obtaining trastuzumab-expressing CHO-K1 cell pools and mini-pools

We first obtained the transfer plasmids bearing the genes encoding for heavy chain (HC) and light chain (LC) of trastuzumab (Fig. 1a). Next, HC gene was cloned into the bicistronic vector pLV-CMV-IRES-Neo (Fig. 1b). Then, we produced LVs in HEK-293T cells to transduce CHO-K1 cells previously adapted to grow in chemically defined, protein-free media and suspension culture. The presence of antibody in the cell culture supernatants of transfected cells was verified by ELISA (data not shown). Two different strategies were followed in parallel (Fig. 2). Strategy 1: based on early screening, generated 19 cell mini-pools: 10 cell mini-pools from co-transduction ratio (400:400) (MOI-LC: MOI-HC) and 9 cell mini-pools from co-transduction ratio (600:200) (MOI-LC: MOI-HC). Strategy 2: rendered two different cell pools, for each co-transduction ratio. All these producing cells were cultured for at least 3 weeks in suspension and shaking conditions in CP-CHO-CB5-FBS medium and showed good growth rate and cell viability above 90%.

**Fig. 3:**
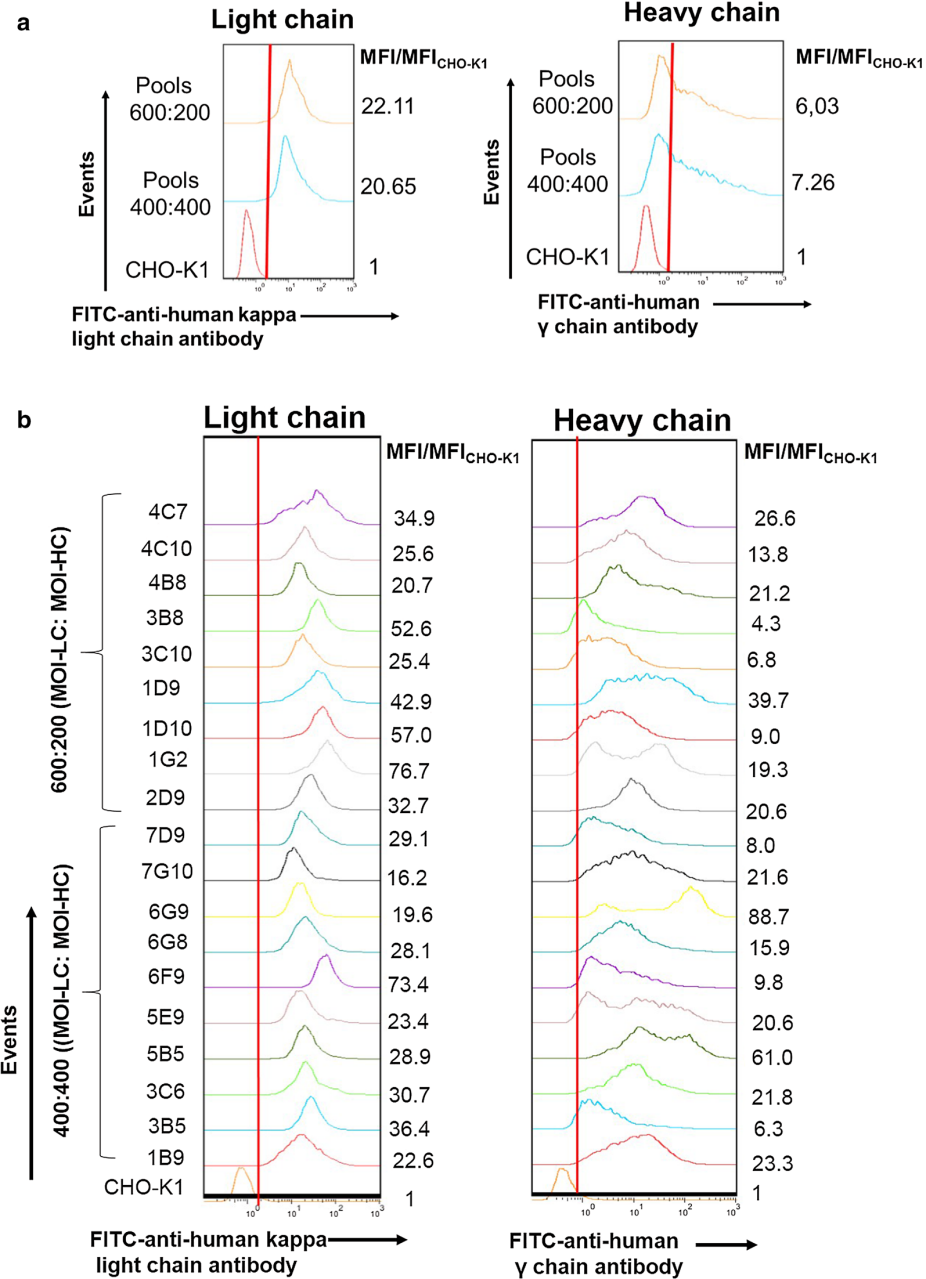
Assessment of intracellular light and heavy chain polypeptides by flow cytometry. **a** cell pools. **b** cell mini-pools. Intracellular light and heavy chains polypeptides of ethanol fixed cells were measured using a FITC-labeled goat anti-human kappa light chain antibody and a FITC-labeled goat anti-human IgG (γ chain specific) antibody, respectively. MFI/MFI_CHO-K1_: mean fluorescence intensity of trastuzumab-expressing cells compared to the mean fluorescence intensity of CHO-K1 wild type cells ratio

We next evaluated the antibody production to select the best candidates to be used in further steps of limiting dilution cloning. First, we assessed cell population homogeneity through measurements of intracellular LC and HC polypeptides by flow cytometry. The mean fluorescence intensity of trastuzumab-expressing cells compared to the mean fluorescence intensity of CHO-K1 wild type cells ratio (MFI/MFI_CHO-K1_) was used for this analysis. Figure 3 shows that all trastuzumab-expressing cells present a homogeneous expression of intracellular LC polypeptide and MFI/MFI_CHO-K1_ values with a coefficient of variation of 49.88% (CV = ratio of standard deviation to the mean). In the case of intracellular HC polypeptide, heterogeneous expression is observed with MFI/MFI_CHO-K1_ values showing a CV of 94.12%. Most cell mini-pools present higher MFI/MFI_CHO-K1_ values related to intracellular HC polypeptide than cell pools (Fig. 3), which means that cell mini-pool populations obtained using the early screening strategy, are more enriched with trastuzumab-expressing cells.

We did not observe significant differences in the intracellular expression of LC or HC polypeptides between cell pools and mini-pools in cells infected with different ratios of LVs for LC and HC (Fig. 3).

**Fig. 4:**
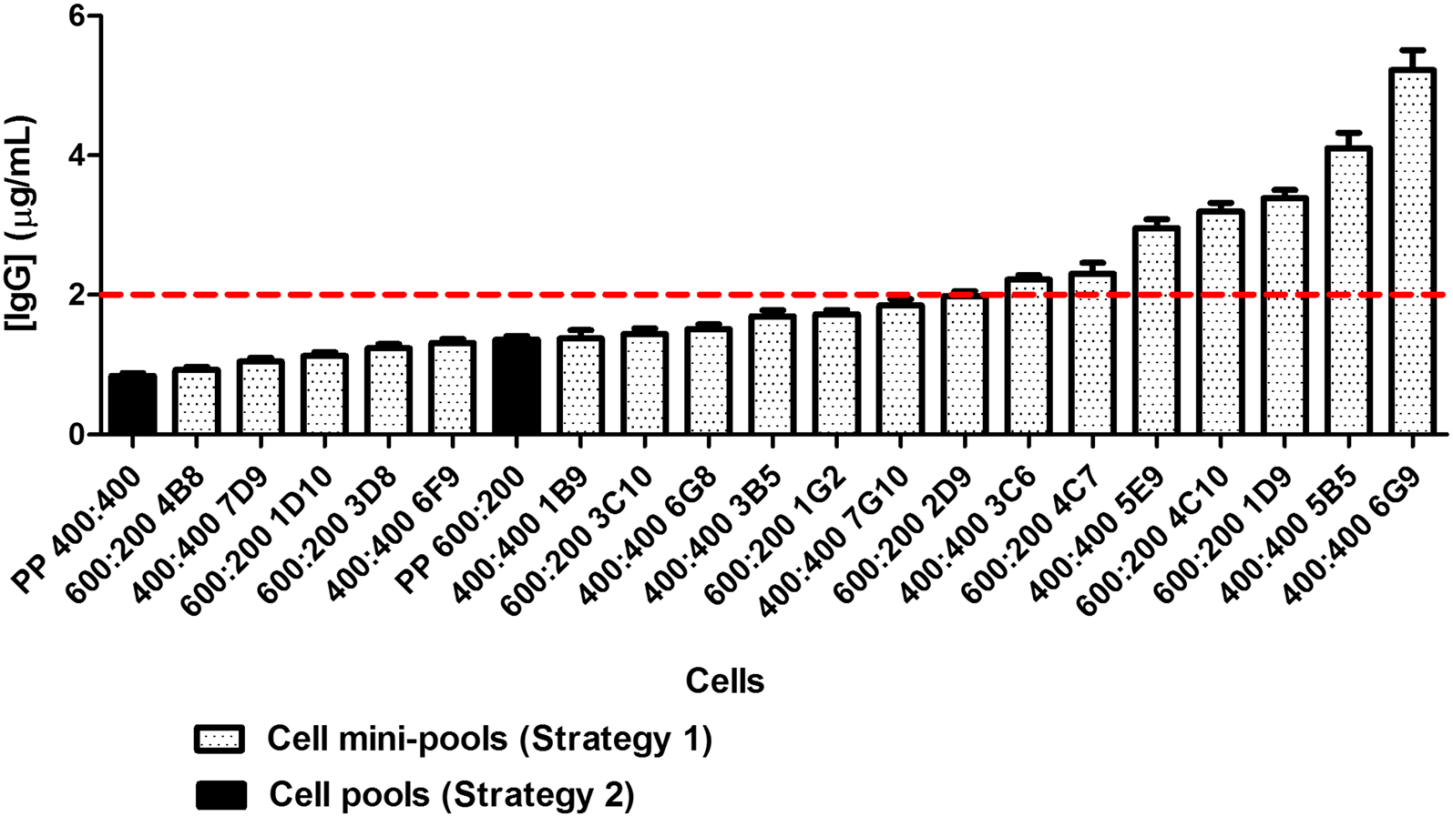
Evaluation of IgG expression levels of cell pools and mini-pools in 24 well plate assay. Producing cells were seeded in 24 well plate, at 0.4 x 10^6^ cells/well in 1 mL of CP-CHO-CB5-FBS medium. The experiment was performed in triplicates. Plates were incubated at 37 °C in the presence of 5% CO_2_. The concentration of IgG in cell culture supernatants after 10 days, was measured by ELISA (whole antibody detection). To quantify the expression levels, a standard curve was made using known amount of trastuzumab. The data correspond to mean ± SD. The discontinuous line indicates the average level of IgG for the evaluated supernatants

IgG levels were also measured in the supernatant from cells culture in 24 well plate with CP-CHO-CB5-FBS medium for 10 days, in order to choose the best expressing mini-pools or pools. The average level of IgG in the cell culture supernatant from the 21 cell groups evaluated was around 2 μg/mL, and only in seven cases was the concentration of IgG above this value (Student’s T test). These seven were mini-pools obtained by the early screening strategy and four of them were obtained from (400:400) (MOI-LC:MOI-HC) co-transduction ratio (Fig. 4).

**Fig. 5:**
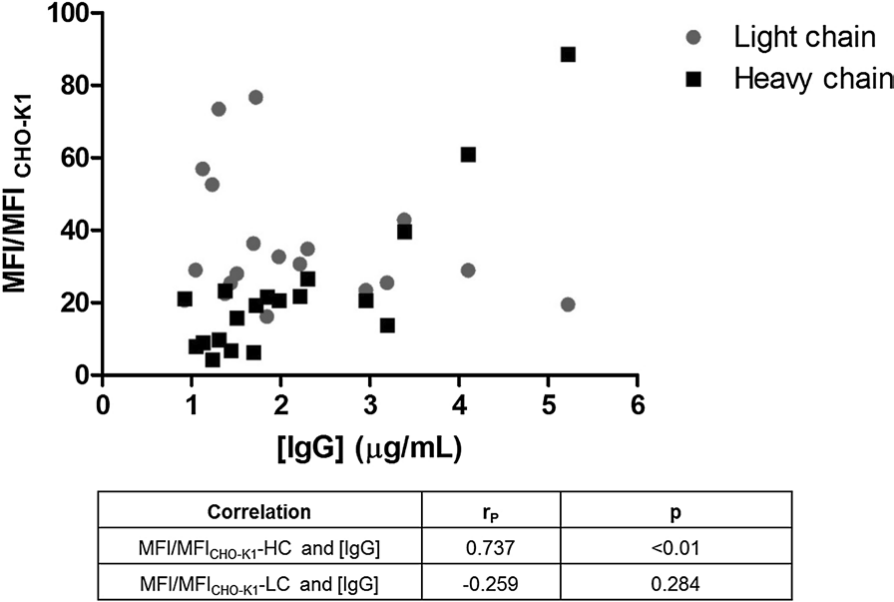
Correlation between intracellular antibody polypeptides content and concentration of IgG in cell culture supernatants. Cell mini-pools were seeded in 24 wells plate in CP-CHO-CB5-FBS medium. After 10 days concentration of IgG in cell culture supernatants was measured by ELISA. Intracellular HC and LC polypeptides of ethanol fixed cells were assessed by flow cytometry. MFI/MFI_CHO-K1_: mean fluorescence intensity of trastuzumab-expressing cells compared to the mean fluorescence intensity of CHO-K1 wild type cells ratio. The correlation between variables was computed using a parametric test based on Pearson correlation

A positive correlation was found between MFI/MFI_CHO-K1_ values of intracellular HC polypeptide content and concentration of IgG in 24 well plate of cell mini-pools (r_P_ = 0.737 and p < 0.01) (Fig. 5). In the case of intracellular LC polypeptide content a correlation with the secreted antibody (r_P_ = − 0.259 and p = 0.284) was not observed.

### Obtaining trastuzumab-expressing CHO-K1 cell oligoclones and clones

Taking into consideration the highest MFI/MFI_CHO-K1_ values of intracellular HC polypeptide content and IgG concentration in the 24 well plate assay, we selected minipools 5B5 and 6G9 originating from the early screening strategy (400:400, MOI-LC: MOI-HC) and adapted to grow in CP-CHO-CB5-FBS, for further cell cloning steps. In order to enrich the population of antibody producing cells and increase the probability to pick high producing clones, we next performed a cell cloning at 10 cells/well in 96 well plate. The 24 oligoclones with the highest IgG expression levels in 96 well plate, were selected and further cultivated in suspension and shaking culture in CP-CHO-CB5-FBS medium. Six oligoclones were obtained from parental cell mini-pool 6G9 and 18 from parental cell mini-pool 5B5. Flow cytometry analysis showed that all oligoclones presented a homogeneous expression of intracellular LC polypeptide and less variable MFI/MFI_CHO-K1_ values (CV of 28.22%) while being more heterogeneous for intracellular HC polypeptide content (CV of 50.56%) (data not shown).

We also measured IgG levels through a 24 well plate assay in CP-CHO-CB5-FBS medium and static culture (data not shown). The average concentration of IgG in cell culture supernatant was around 7.9 μg/mL and 11 oligoclones were above this value (Student’s T test). Seven oligoclones were selected, including the five with higher expression levels in a 24 well plate assay, and further adapted to grow directly in CP-CHO-CB5 medium without serum supplementation. Twenty-five cm^2^ T-flasks were used to simulate small-scale bioreactors that could provide more precise information about the expression potential of these producing cells. The highest secreted IgG levels were exhibited by oligoclones 10B10 (56.1 μg/mL) and 2F3 (51.5 μg/mL) (from parental cell mini-pool 5B5) and 10D3 (54 μg/mL) (originated from parental cell mini-pool 6G9).

**Fig. 6:**
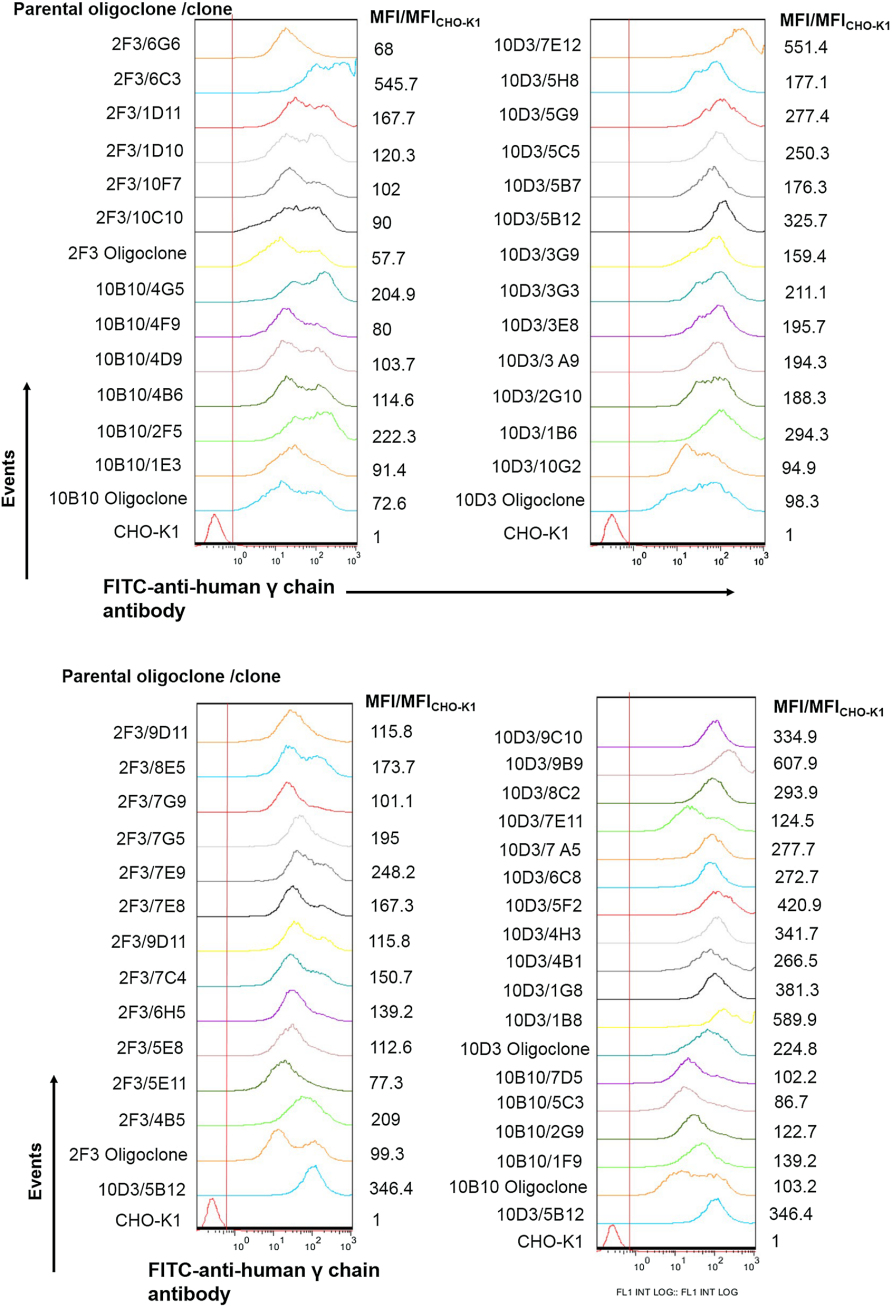
Assessment of heavy chain polypeptide content of cell clones by flow cytometry. Intracellular heavy chain polypeptides of ethanol fixed cells were measured using a FITC-labeled goat anti-human IgG (γ chain specific) antibody. MFI/MFI_CHO-K1_: mean fluorescence intensity of trastuzumab-expressing cells compared to the mean fluorescence intensity of CHO-K1 wild type cells ratio

**Fig. 7:**
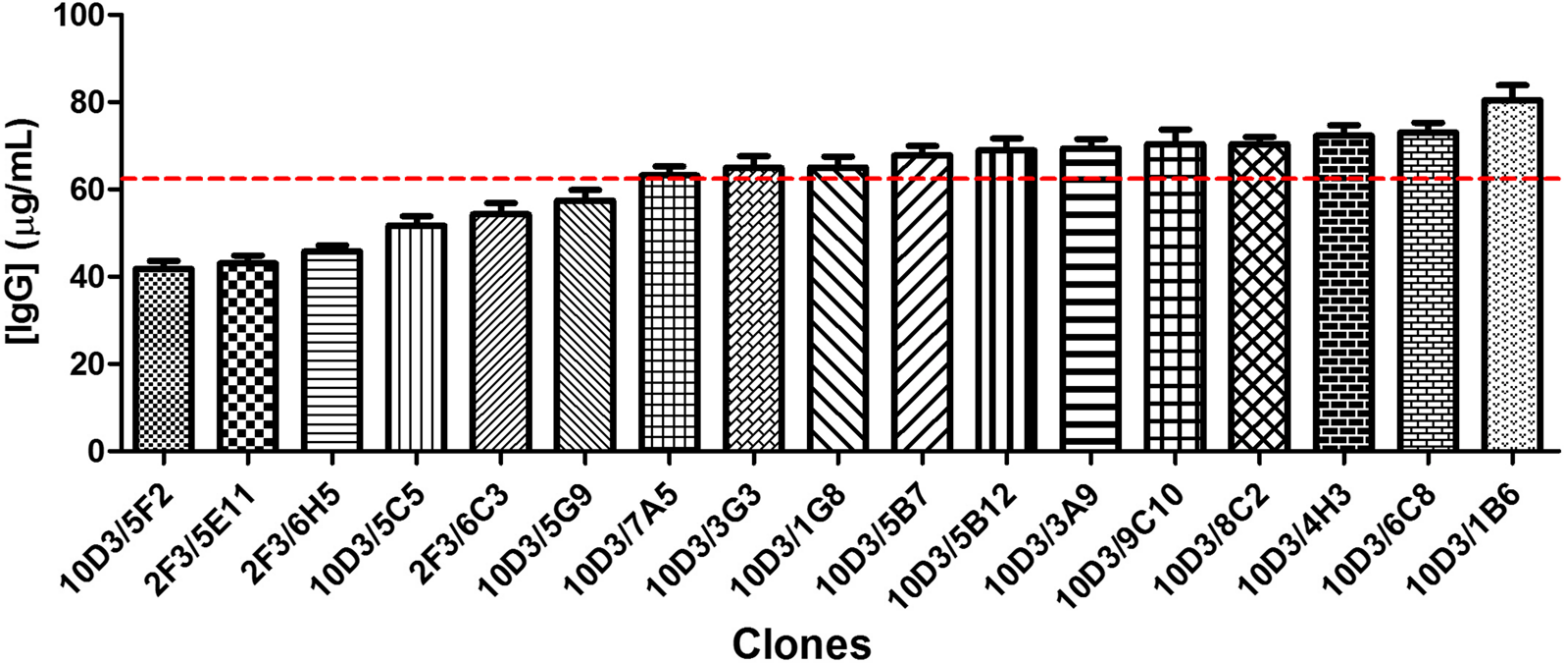
IgG expression levels of trastuzumab-expressing clones in chemically defined, protein-free medium and shaking T-flasks. Producing cells were seeded in 25 cm^2^ T-flasks at 0.4 x 10^6^ cells/well in 10 mL of CP-CHO-CB5 medium. The experiment was performed in duplicates. T-flasks were incubated at 37 °C in the presence of 5% CO_2_ in vertical position and after 10 days cell culture supernatant was removed to quantify human IgG-expression levels by ELISA. To quantify the expression levels, a standard curve was made using known amount of trastuzumab. The data correspond to mean ± SD. The discontinuous line indicates the average level of IgG for all the evaluated supernatants

After a step of limiting dilution cloning at low cell density (0.5 cells/well) in DMEM/F12-FBS, 52 clones were obtained and re-adapted to grow directly in suspension culture and chemically defined, protein-free medium. Then, we assessed only intracellular HC polypeptide content by flow cytometry because in previous experiments it showed more variability and, therefore gives more information about cell population homogeneity. Figure 6 shows that some clones presented a homogeneous expression of intracellular HC polypeptide while others, two subpopulations. Afterwards, clones with a homogeneous cell population were selected. Seventeen clones were selected and their levels of expression in suspension culture and chemically defined, protein-free medium were assessed using the T-flasks approach previously mentioned. The average level of IgG in the cell culture supernatant in this experiment was around 62.8 μg/mL and 10 clones were above this value (Student’s T test) (Fig. 7).

### Batch culture

**Fig. 8:**
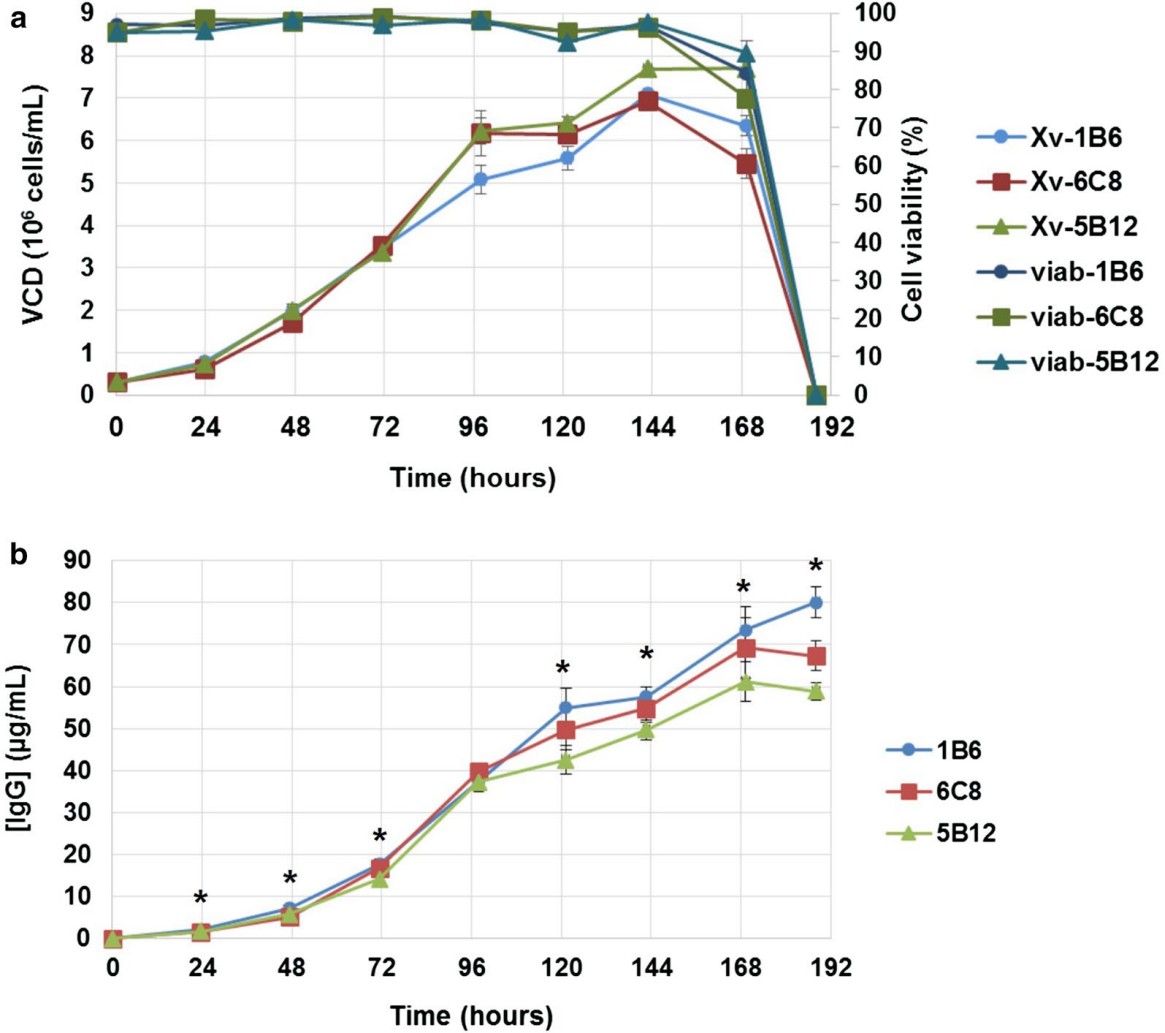
Batch culture for selected trastuzumab-expressing clones in chemically defined, protein-free medium and shaking culture. **a** Growth profile. **b** Titer of clones. Shaking Erlenmeyer flasks with 60 mL of CP-CHO-CB5 medium were inoculated with 0.3 x 10^6^ cells/mL. The experiment was performed in duplicates. These flasks were incubated at 37 °C in the presence of 5% CO_2_ and shaking culture (120 rpm) (Infors HT, Switzerland). VCD: viable cell density. All the clones are originated from parental cell oligoclone 10D3. The data correspond to mean ± SD. (*) indicates significant difference between means of almost two clones

Clones 6C8, 1B6 and 5B12 with expression levels above 62.8 μg/mL and no macroscopic cell aggregates after 10 days of culture in shaking T-flaks, were further characterized by batch culture using shaking Erlenmeyer flasks. The growth profiles of these clones were very similar, with cell viability above 90% after 6 days; however, cells died abruptly from day 7 to 8 (Fig. 8). Clone 5B12 showed the higher values of viable cell density (VCD) and time integral for viable cell concentration (IVCC), while those parameters were practically the same for clones 6C8 and 1B6. Specific productivity was very similar among the three clones. Significant differences in concentration of IgG were observed at the end of the experiment. Table 1 summarizes productivity and growth characteristics of these three clones in batch culture.

**Table 1:** Productivity and growth characteristics of selected trastuzumab-expressing CHO-K1 clones in batch culture (chemically defined, protein-free medium and shaking culture)

Clones	VCD_max_ (10^6^ cells/mL)	IVCC (10^8^ cells*h/mL)	μ_max_ (h^−1^)	Duration (days)	[IgG]_max_ (μg/mL)	q_IgG_ (pcd)
5B12	7.7	811.59	0.0237	8	58.88 ± 3.55	3.8
6C8	6.925	729.97	0.0247	8	69.01 ± 2.24	3.86
1B6	7.1	723.01	0.0196	8	80.08 ± 3.68	4.05

*VCD*_*max*_ maximum viable cell density, *IVCC* time integral of viable cell concentration, *μ*_*max*_ maximum growth rate, *q*_*IgG*_ specific productivity

### Western blotting and HER2-recognition assay

**Fig. 9:**
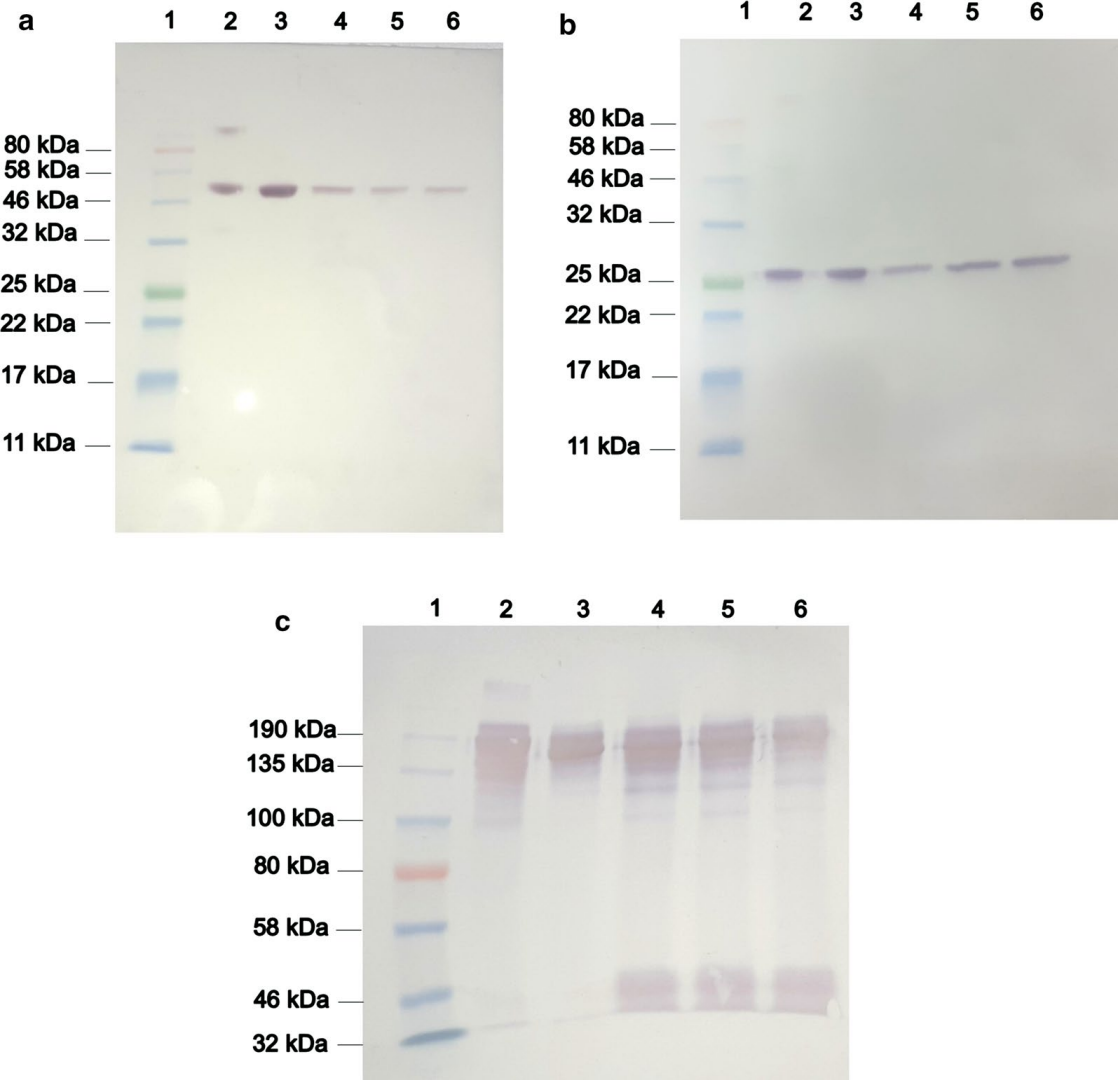
Analysis of biosimilar candidate to trastuzumab in cell culture supernatant by Western blotting. Cell culture supernatants of cells growing in chemically defined, protein-free medium and shaking culture were loaded in 12% SDS-PAGE/reducing conditions (**a** and **b**) and 7.5% SDS-PAGE/non-reducing conditions (**c**). Immunodetection of heavy chain (**a**) was performed using an AP-conjugated goat anti-human IgG (γ chain specific) antibody. Immunodetection of light chain (**b**) and antibody whole molecule (**c**) was performed using a HRP-conjugated goat anti-human kappa light chain antibody. 1: Pre-stained molecular weight marker (NEB, UK). 2: biosimilar to trastuzumab obtained from NS0 cells (2.5 μg) (named 5G4). 3: Commercial trastuzumab (2.5 μg). 4: Cell culture supernatant of 5B12 clone. 5: Cell culture supernatant of 1B6 clone. 6: Cell culture supernatant of 6C8 clone

The cell culture supernatants of the selected clones (1B6, 6C8, 5B12) grown in batch culture, were used to verify protein identity by western blotting. The western blotting results, under reducing conditions, corroborated the expected migration pattern for heavy (Fig. 9a) and light (Fig. 9b) chains, corresponding to 50 kDa and 25 kDa, respectively. Under non-reducing conditions, the recombinant antibody exhibited a similar immunoreactive profile compared with commercial trastuzumab, exhibiting similar molecular weights (Fig. 9c). Moreover, some bands around 50 kDa were observed, possibly corresponding to LC dimers.

**Fig. 10:**
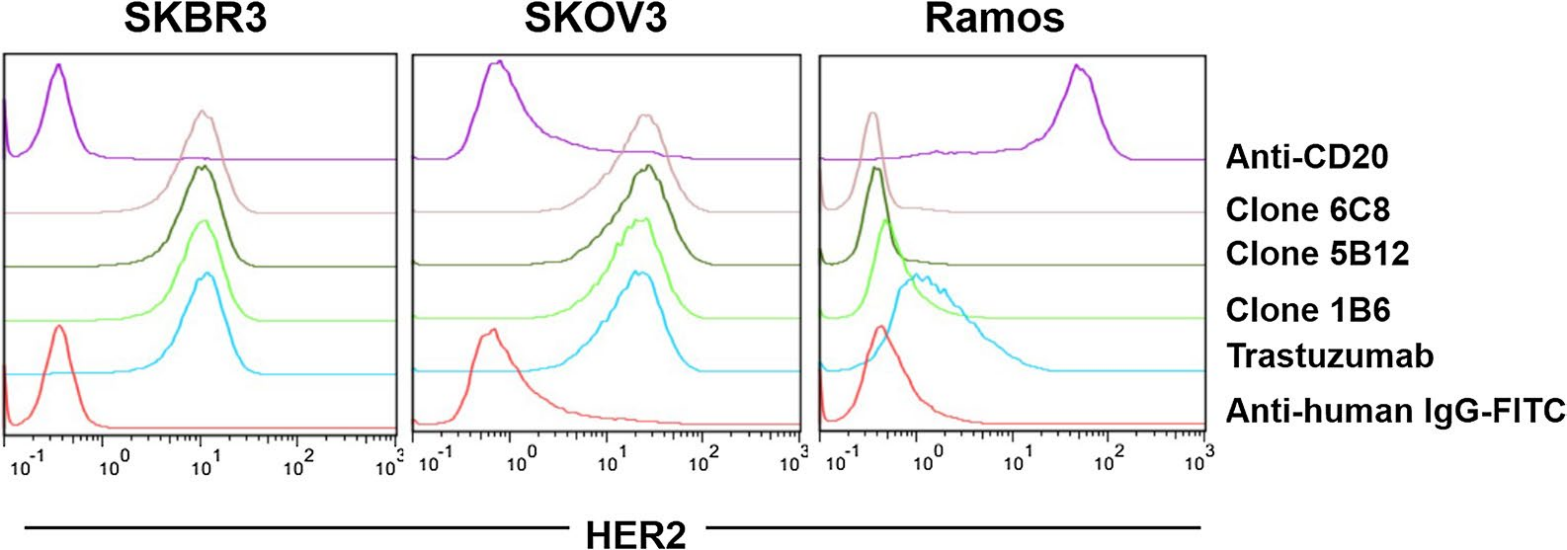
Recognition of HER2-expressing tumor cell lines. SKOV3, SKBR3 and Ramos cell lines were stained with 10 μg/mL of produced antibody contained in cell culture supernatants of 1B6, 6C8 and 5B12 clones, followed by a FITC-labeled rabbit anti-human IgG antibody. Commercial trastuzumab was used as positive control and cell culture supernatant of anti-CD20 expressing clone, as isotype matched control. Ramos cells (CD20 +) were used as control of non-HER2 expressing cells. Results are representative of two independent experiments

Finally, we evaluated the binding specificity of the produced antibody using flow cytometry. The HER2 binding assay demonstrated that the recombinant antibody contained in cell culture supernatants of clones 1B6, 6C8 and 5B12 can recognize HER2 overexpressed in SKOV3 and SKBR3 cells in a similar way as commercial trastuzumab (Fig. 10). In addition, there was no recognition of Ramos cells (HER2-).

## Discussion

This work focused on the generation of biosimilar to trastuzumab-expressing CHO-K1 cells through a screening and selection strategy. The recombinant CHO-K1 cells were obtained using LVs. One advantage of LVs, is the possibility to influence the transgene copy number and/or the percentage of infected cells by adjusting the virus to cell ratio (Gödecke et al. 2018; Oberbek et al. 2011). In this way, increased MOI values could mean higher copy number of the DNA of interest inside the genome of host cells; and therefore, higher levels of target protein expression. On the other hand, it has been reported that the ratio of the copy number of the genes for LC and HC can affect the productivity of the recombinant cell lines and the quality of the antibody (Chusainow et al. 2009; Ho et al. 2013; Jiang et al. 2006; Schlatter et al. 2005). Some authors have shown that an excess of LC polypeptide can improve productivity and decrease protein aggregation (Ho et al. 2012; Ho et al. 2013; Lee et al. 2009; Schlatter et al. 2005). Thus, it is possible to improve expression levels by optimizing gene doses (Jiang et al. 2006). Taking into account both advantages, in this work, higher values of MOI and two different LC to HC ratios were used: (MOI-LC:MOI-HC) at (400:400) and at (600:200). However, expected levels of expression were not obtained and no significant differences in intracellular content of LC and HC polypeptides were observed in cell mini-pools or pools.

Once cells are transfected, subsequently one or more selection steps are applied to selectively kill cells that did not stably integrate the expression vector into their genome (Agrawal et al. 2012; Hacker and Balasubramanian 2016; Jostock 2011). Those selection steps could either be performed on the entire transfected population (pools) or on small pools (mini-pools) of cells distributed in 96 well plates at around 100-2000 cells/well (Agrawal et al. 2012; de la Cruz Edmonds et al. 2006; Kober et al. 2013; Noh et al. 2018). In this current study, both strategies were used. The cell populations obtained were assessed by measuring the levels of secreted antibody and the intracellular content of LC and HC polypeptides by flow cytometry. The results showed that the early screening strategy (or cell mini-pool approach) resulted in recombinant CHO-K1 cell populations with higher enrichment of IgG-expressing cells.

Since cell line screening is a time and resources-consuming as well as a labor-intensive process, several research approaches have focused on finding methods for identifying high-producing clones with good product quality early in the cell line development screening process. Some researchers have found positive correlations between specific productivity and HC mRNA levels in antibody producing CHO cells (Edros et al. 2013; Jiang et al. 2006; Lee et al. 2009) or IgG titers and HC mRNA in myeloma cells (Dorai et al. 2006). However, a disadvantage of these assessments is that they do not take into account or provide information on the heterogeneity of the population. They only describe the average characteristics of the cells in a cell line. In contrast, the measurement of intracellular HC and LC polypeptides by flow cytometry offers a direct method of assessing the antibody content of single cells within a cell culture (Roy et al. 2017). Edros et al. (2013) found an association between specific productivity HC intracellular polypeptide using flow cytometry. Furthermore, Roy et al. (2017), measured intracellular HC polypeptide content to identify the best antibody-expressing NS0 cell clones in early stages of the selection process. They observed a positive correlation between intracellular HC polypeptide content and specific productivity, and also final volumetric productivity (Roy et al. 2017). In agreement with to these observations, our results shown a positive correlation between MFI/MFI_CHO-K1_ values of intracellular HC polypeptide content and IgG titers in 24 well plate assay of cell mini-pools. Our results support that intracellular HC polypeptide content measurements by flow cytometry is a useful tool to identify the higher expressing candidates in early stages of process. Moreover, this flow cytometric tool significantly reduces the timeline and efforts towards final clone selection (Roy et al. 2017).

The measure of intracellular polypeptide content is also an effective tool to evaluate the homogeneity and stability of producing cell populations (Dorai et al. 2012; Krebs et al. 2018). Herein, we also measured intracellular LC and HC polypeptides contents for IgG-expressing oligoclones and clones (data not shown). The results show that in the case of intracellular LC content, homogeneous and uni-modal distributions were observed. In addition, all these cell populations (cell pools, mini-pools, oligoclones and clones) presented different expression levels. However, cell pools and mini-pools show a more heterogeneous distribution of intracellular HC polypeptide content and variable MFI/MFI_CHO-K1_ values compare to cell oligoclones and clones. Indeed, the clones presented a homogeneous or bi-modal distributions related to intracellular HC polypeptides content. Several authors have described that many Ig-producing cell lines in nature secrete an excess of LC which is not associated to HC, and this have been observed the biopharmaceutical industry for the generation of IgG-expressing cell lines (Krebs et al. 2018). This phenomenon can be explained since the glycosylation of HC apparently blocks its transduction (Bergman et al. 1981; Krebs et al. 2018). Therefore, the internal measurement of the HC portion of an antibody therapeutic protein, proved empirically to be the most reliable representative of ultimate intact protein expression levels (Krebs et al. 2018).

Several authors have found a decrease in antibody titers over time is associated with the rise of secondary cell population with a low intracellular HC polypeptide content, and therefore, with the instability of these cell lines expressing recombinant antibodies. So, this methodology could be implemented to identify which cell clones will have a better behavior in the production setting (Dorai et al. 2012; Krebs et al. 2018). Taking into account these experience, we used homogeneous distribution as a criterion for selection of cell clones for further analyzes.

The use of CHO-K1 cells pre-adapted to grow in protein-free media and suspension culture as the cell host, considerable decreased the time and efforts needed to re-adapt the recombinant CHO cells to these cell culture conditions (Kim et al. 2012). Indeed, we cultured recombinant cells directly in a chemically defined, protein-free medium, after a step limiting dilution cloning with 5% FBS, and cells showed good growth and cellular viability above 90%. So, a final step of adaptation to chemically defined, protein-free media and suspension culture was not needed.

According to Porter et al. (2010) a challenge in the selection of cell lines destined for cGMP manufacture is that the behavior of a cell line early on in development may not reflect the behavior of that cell line in the final production process (Porter et al. 2010). Here, we used the shaking 25 cm^2^ T-flasks approach to screen a great number of candidates under conditions closer to a final production process. Other example of a less expensive alternative to microbioreactors is the use of 24 well deep plates to evaluated cell lines in suspension and stirred culture (Mora et al. 2018). Moreover, batch culture in shaking Erlenmeyer flasks for producing clones selected from shaking 25 cm^2^ T-flasks, showed similar values of IgG expression levels compared to 25 cm^2^ T-flasks approach.

The highest expression levels reached during shaking 25 cm^2^ T-flasks assays or batch culture are 3 to 4 times lower than highest results reported in the literature for recombinant proteins-expressing CHO cells using LVs (Gaillet et al. 2010; Oberbek et al. 2011). A comparison between our batch culture results from trastuzumab-expressing cell clones and other CHO cell clones reported in the literature indicates our expression levels are lower (4-10 times) than expected. For example, LeFourn et al. (2014) generated several trastuzumab-expressing CHO-K1 cell clones through electroporation, selection with puromycin and limiting dilution cloning. The kinetics of these clones were studied by batch culture in 50 mL mini-bioreactors during 7 days. The maximum concentration of IgG was 300-800 μg/mL, average specific productivity was 22.8 pcd and maximum viable cell concentration was 2-8 x 10^6^ cells/mL (Le Fourn et al. 2014). An increase of trastuzumab expression levels could be possible through optimization of culture media composition and fermentation conditions (Bandaranayake and Almo 2014; Li et al. 2010; Wurm and de Jesus 2016). Preliminary results related to HER2-recognition capacity of biosimilar to trastuzumab candidate were successful. Nevertheless, further experiments to assess antibody dependent cell-mediated cytotoxicity (ADCC) must be done, as well an extensive analytical characterization such as peptide mapping, whole mass analysis, N-glycan mapping, size variants analysis, among others (Hutterer et al. 2019; Kim et al. 2017).

In conclusion, trastuzumab-expressing CHO-K1 cell lines were generated through a strategy which combined LVs; CHO-K1 cells pre-adapted to chemically defined, protein-free media and suspension culture; a shaking 25 cm^2^ T-flask approach and the assessment of intracellular HC polypeptide by flow cytometry and its potential as early stage predictor of levels of expression. This strategy is very useful when low throughput techniques, such as limiting dilution cloning, are used to select high producing cells and there is no access to automatized technology as CellCelector™ and ClonePix FL™ or even flow cytometric cell sorter instruments or micro-bioreactors. Indeed, IgG-expressing CHO-K1 cells, adapted to chemically defined, protein-free media and suspension culture, could be generated in 2.5–4 months. Therefore, this strategy can be used as a platform for obtaining other biosimilars to monoclonal antibodies in CHO cells. In addition to low expression levels of trastuzumab-expressing CHO-K1 cells generated in this work, we believe that improvements in up-stream steps such as tricistronic vectors, UCOEs, MARs, codon optimization, signal peptide optimization, amplification/selection systems, transfection/transduction procedures; and down-stream steps such as media composition and fermentation condition related to this strategy, it could be possible to reach specific productivities around 20–30 pcd and volumetric productivities above 10 g/L.

## Data Availability

The datasets used and/or analyzed during the current study are available from the corresponding author on reasonable request.

## Abbreviations

CHO: Chinese hamster ovary
HC: Heavy chain
HER2: Human epidermal growth factor receptor 2
IVCC: Time integral of viable cell concentration
LC: Light chain
LVs: Lentiviral vectors
MFI: Mean fluorescence intensity
MOI: Multiplicity of infection
q_IgG_: Specific productivity
VCD_max_: Maximum viable cell density
μ_max_: Maximum growth rate
